# Genetic and Environmental Factors in Ageing and Age-Related Disease

**DOI:** 10.3390/genes13030396

**Published:** 2022-02-23

**Authors:** Karen A. Mather

**Affiliations:** Centre for Healthy Brain Ageing, Discipline of Psychiatry and Mental Health, Faculty of Medicine, University of New South Wales (UNSW), Sydney, NSW 2031, Australia; karen.mather@unsw.edu.au

Globally, the population is growing older. A better understanding of the contributions of genetic, epigenetic and environmental factors to human ageing will ultimately facilitate strategies to promote health in older adults. This Special Issue of *Genes* provides examples of the high-quality research undertaken in this field.

Li and colleagues [1] explore the role of formaldehyde in age-related cognitive impairment. Although formaldehyde is often thought of as a toxic chemical, endogenous formaldehyde has many important roles, including participating in the one-carbon cycle and epigenetic regulation. The authors posit the hypothesis that endogenous formaldehyde is involved in memory formation/loss through the methylation/demethylation of DNA, RNA and histones.

In their thought-provoking article, Larocca et al. [2] discuss the regenerative properties of immortal germline (‘clock-free’) versus soma cells that are ‘clock-bound’, which lose regenerative capacity as the cellular clocks (e.g., telomeric) begin counting down. The authors discuss that the reprograming of cellular clocks using germline factors may lead to future therapies to slow down ageing, or even reverse it, and extend the health span.

Sarcopenia is linked to reduced mobility, disability, loss of independence and falls in older adults. Lee and Neppl review the major anabolic and catabolic pathways that regulate skeletal mass and strength [3].

Examining a Japanese sample of the oldest old, Sasaki et al. [4] examine the genetics of alcohol consumption, finding that a missense variant in the aldehyde dehydrogenase gene was associated with current and quitting alcohol drinking behaviours. 

The major single genetic risk factor for late-onset Alzheimer’s disease is the apolipoprotein (*APOE*) ε4 allele. In addition to optimising APOE sequencing, Gonzalez et al. [5] found allele differences in the ε4 and ε2 alleles in late-onset Alzheimer’s disease cases between Spanish and Portuguese subsamples, highlighting the importance of considering different ancestries and diverse populations when undertaking these studies. 

Smoking is a key lifestyle factor which is linked to premature aging, accelerated epigenetic ageing and early mortality. It has been linked to changes in DNA methylation sites, some of which have been reported to revert during smoking cessation. Philibert et al. [6] tested this hypothesis. Their results showed a reversion of the DNA methylation site over a 90-day quitting period. The authors conclude that monitoring the status of this DNA methylation site may be a useful tool for motivating smokers to quit and the monitoring of smoking cessation behaviours.

The origin of DNA double-strand breaks and its impact on ageing is discussed in a paper by Kuma Tripathy et al. [7]. The authors suggest that the genomic integration of cell-free chromatin particles released from dying cells into healthy cells can result in double-stranded breaks, faulty repair, apoptosis and the release of more cell-free chromatin, which is accompanied by the activation of inflammatory cytokines. They propose that double-strand breaks and inflammation activation may be a cause of ageing.

In vivo studies suggest that stress and glucocorticoid stress hormones are associated with shorter telomere length, although experimental evidence is lacking. In a brief report, Zannas and colleagues [8] address this shortcoming by reporting that prolonged exposure to glucocorticoid stress does not accelerate telomere shortening in human fibroblasts.

In conclusion, this Special Issue demonstrates the depth and breadth of research in ageing, indicating the many possibilities for future research and advances in this field.

## References

[1] Li T., Wei Y., Qu M., Mou L., Miao J., Xi M., Liu Y., He R. (2021). Formaldehyde and De/Methylation in Age-Related Cognitive Impairment. Genes.

[2] Larocca D., Lee J., West M.D., Labat I., Sternberg H. (2021). No Time to Age: Uncoupling Aging from Chronological Time. Genes.

[3] Lee E.J., Neppl R.L. (2021). Influence of Age on Skeletal Muscle Hypertrophy and Atrophy Signaling: Established Paradigms and Unexpected Links. Genes.

[4] Sasaki T., Nishimoto Y., Hirata T., Abe Y., Takebayashi T., Arai Y. (2021). *ALDH2* p.E504K Variation and Sex Are Major Factors Associated with Current and Quitting Alcohol Drinking in Japanese Oldest Old. Genes.

[5] González R.D., Gomes I., Gomes C., Rocha R., Durães L., Sousa P., Figueruelo M., Rodríguez M., Pita C., Hornero R. (2020). *APOE* Variants in an Iberian Alzheimer Cohort Detected through an Optimized Sanger Sequencing Protocol. Genes.

[6] Hilibert R., Mills J.A., Long J.D., Salisbury S.E., Comellas A., Gerke A., Dawes K., Vander Weg M., Hoffman E.A. (2020). The Reversion of cg05575921 Methylation in Smoking Cessation: A Potential Tool for Incentivizing Healthy Aging. Genes.

[7] Tripathy B.K., Pal K., Shabrish S., Mittra I. (2021). A New Perspective on the Origin of DNA Double-Strand Breaks and Its Implications for Ageing. Genes.

[8] Zannas A.S., Kosyk O., Leung C.S. (2020). Prolonged Glucocorticoid Exposure Does Not Accelerate Telomere Shortening in Cultured Human Fibroblasts. Genes.

